# Effects of wingwave^®^ on athletes’ wellbeing and fluidity of gaze behavior

**DOI:** 10.3389/fpsyg.2023.1010063

**Published:** 2023-02-23

**Authors:** Frank Peter Gadso Weiland, Benjamin Noël, Stefanie Klatt

**Affiliations:** Department of Cognitive and Team/Racket Sport Research, Institute of Exercise Training and Sport Informatics, German Sport University Cologne, Cologne, Germany

**Keywords:** EMDR, saccades, eye tracking, short-term coaching, team sport

## Abstract

**Introduction:**

The wingwave^®^ method combining a muscular test and elements of eye movement desensitization and reprocessing has been found to successfully reduce anxiety and improve relaxation in people. However, it is still unclear how exactly its application improves individual wellbeing (though it is assumed to be related to changes in gaze patterns) and if it works for team sport athletes.

**Method:**

To test both, team sport athletes who had reported a problem with a team sport aspect were randomly assigned to an experimental group and a control group. The experimental group members were individually coached by a wingwave^®^ coach once while the other participants watched a tennis match instead.

**Results:**

Results showed that athletes in the wingwave^®^ group benefited from the coaching as their individual perception of their problems improved. These improvements were associated with a decrease of catch-up saccades in a visual object-tracking task conducted before and immediately after coaching.

**Discussion:**

This points to wingwave^®^ interventions affecting gaze behavior and consequently wellbeing of team sport athletes.

## Introduction

Sports and especially team sports are characterized by pressure on the athletes to perform and often make decisions and act within a split second (e.g., Vilar et al., 2013). Therefore, when evaluating performance, it is important to consider not only the individual physical, tactical, and cognitive prerequisites, but also negative thoughts regarding their sporting environment, events, and experiences during their competitions. When creating an environment in which athletes can perform best, having negative emotions is associated with underperformance (Barsade and Gibson, 2012; Hill and Shaw, 2013). In sport psychology there is a large body of research looking at the direct effects of stress on performance (Arnold and Fletcher, 2021), and there are also many studies looking at how best to regulate or minimize stress and pressure to perform (Ong and Chua, 2021). There is already evidence that different relaxation techniques as meditation or breathing techniques are effective in sports environments (Parnabas et al., 2014).

Most of these techniques require regular use by the athlete, however, there is also a method which, when used infrequently, could bring medium and sometimes, even long-term benefits without an extensive time investment after the initial use. This coaching method which seemed promising based on initial research (Rathschlag and Memmert, 2014), but has not been analyzed extensively in the sport context, is the wingwave® method. To fully understand this intervention technique, it is important to look at its crucial core first, which is EMDR (Eye movement desensitization and reprocessing).

EMDR was developed and first described by Shapiro (1989) and has been found to be an effective therapeutic intervention for post-traumatic stress disorder (PTSD), childhood trauma (i.e., in adults and children) as well as symptoms of anxiety and depressions (e.g., Chen et al., 2018; Wilson et al., 2018). In general, EMDR has been shown to be helpful after stressful life experiences (Shapiro, 2018). In a therapy session based on EMDR, eight phases are executed sequentially. The crucial phase is the so-called desensitization-phase, in which the previously identified issue (i.e., in the other phases) is focused on. This issue, being a traumatic moment in the client’s past, is then focused on by the client, keeping the awareness of the visual image, rational or emotional thoughts, body cognizance or auditory stimuli that are remembered. During this process, brief sets of quick bilateral stimulations are prompted by the therapist until the client’s perception of the thoughts or associated physical sensations improve (Landin-Romero et al., 2018). The bilateral stimuli originate from the therapist’s fingers moving from side to side while the client follows this procedure with his or her eyes. The stimuli may also be auditory or tactile as a complement to the finger movement or instead of it (Benor et al., 2017). EMDR has also been found to be effective in non-therapeutic contexts. Maxfield and Melnyk (2000) could show that test anxiety could be reduced by a single session of EMDR. Brooker (2018) found that EMDR decreased anxiety and increased performance confidence in music performers.

The wingwave® method exclusively focusses on clients in non-clinical environments, i.e., coaching-settings. Emotional blockades at work, in school or in sports, are typical causes of clients looking for non-therapeutic help in a wingwave® coach. The method’s concept is to combine the bilateral stimulation part described above with a muscular test, during which the clients form a “ring” with their index finger and thumb and the coach tries to open this “ring” with both hands (cf. Omura, 1985). Whenever the client experiences negative emotions, the ring is easier to open than in emotionally neutral or positive states. Wingwave® coaches use the muscular test to narrow down singular events in the past of the client – like traumatic memories in classical EMDR – with a manual (cf. Weiland et al., 2021). In the beginning of each coaching, the client defines the topic, and the coach uses the muscular test to find hints that lead to the specific corresponding memory concerning time (e.g., age 1–10 and age 11–20) setting (e.g., school, work, and holiday) or emotion (e.g., anxiety, anger, helplessness). After calling out a word, the coach shortly thereafter tests the strength of the client’s “ring.” If it remains closed while the coach tries to open it, the coach tries the next suitable word and pulls again (e.g., “age 11–20” after “age 1–10”). Whenever the “ring” opens, the word just said by the coach is a hint to the stressful memory. As soon as all manually defined hints to narrow down the stressful memory have been found, the clients are asked to be aware and share their bodily sensations, thoughts etc. concurrent with the experienced emotion connected to the memory. The coach then executes bilateral stimulation as in EMDR in brief sets until the client reports a neutral or positive feeling towards the topic defined for the coaching-setting and the “ring” can no longer be opened by the coach after naming the coaching topic for the client.

Weiland et al. (2021) organized a study, in which schoolchildren of age 11 and 12 years were randomly assigned to either an experimental or a control group. In both groups, the children were tested three times within 8 to 10 weeks concerning their school anxiety, manifested anxiety, school dislike and concentration performance. The first three were a part of a paper-pencil self-evaluation-inventory, and the concentration performance was administered through an 18-min-math-test. In addition to the self-evaluation-inventories, the subjective feeling of the child towards two self-chosen school subjects was tested all the three times. Between test 1 and 2, the children in the experimental group received three wingwave® coachings with a duration of 60 min each. The control group did not receive any treatment. The results of the study showed that emotional distress among the schoolchildren decreased, and performance increased in the experimental group, which was not the case in the control group. Furthermore, the subjective feeling towards the chosen subjects improved in the experimental group as well as in the control group. Wingwave® coaching, thus, may lead to a less anxious perception of school, or at least aspects of school, and improved concentration among schoolchildren. However, given that wingwave® coachings were shown to be effective in various environments (e.g., Rathschlag and Memmert, 2014; Weiland et al., 2021, 2022) it seems fruitful to further analyze if it can also be applied to reduce sport related issues in team sport athletes.

Furthermore, it is still not completely clear in what way the wingwave® method works exactly. Kapoula et al. (2010) showed that application of EMDR is associated with more fluid pursuit eye movements in a standard laboratory task. This means that after the application of EMDR, participants made less catch-up saccades while visually tracking a moving dot on a computer screen than prior to EMDR sessions. Thus, participants’ gaze was less often lagging behind the moving dot making saccades to catch up more often unnecessary. van den Hout et al. (2001) reported that eye movements influence the vividness and emotional rating of personal memories in a way that negative and positive memories become less extreme. Elofsson et al. (2008) reasoned based on their study analyzing several physiological stress markers (e.g., skin conductance) before and after EMDR treatment that perhaps the application of EMDR leads to an activation of a cholinergic response and inhibition of sympathetic systems. Though, according to the authors, there are several potential theories on EMDR, it seems likely that the observed physiological reactivity has similarities to the REM sleep pattern interventions. Elofsson et al. (2008) also consider it reasonable that gaze behavior or changes to it play an important role in EMDR effects.

All these studies points to the importance of gaze behavior (manipulations) in EMDR application, making it likely that at least some of the effects of wingwave® coachings are also associated with changes of gaze related aspects. Considering the work of Kapoula et al. (2010) we aimed to test if the fluidity of pursuit eye movements is impacted by the application of wingwave® coaching sessions. Therefore, the aims of the current study were twofold. First, we wanted to test if application of wingwave® coaching improve ratings of team sports related issues and, second, we aimed to analyze the relationship between wingwave® coachings and changes of fluidity of gaze in a standardized, visual tracking task.

## Methods

### Participants

Fifty-two sport students (24 male, 28 female; *M* = 23.77 years of age; *SD* = 3.1) took part in the experiment. Participants reported normal or corrected to normal vision. At the time of data collection, participants were regularly active in a team sport with a training frequency of at least three times a week and regularly participated in competitions of their sports (e.g., league games, tournaments). Primary sports included soccer (*n* = 16), handball (*n* = 16), (beach-)volleyball (*n* = 11), field hockey (*n* = 5), and basketball (*n* = 4). Further conditions for inclusion in the study were that participants had not yet gained any experience with the wingwave® method. Sample size requirements were calculated using G*Power 3.1 (Faul et al., 2009), indicating that a sample size of 25 participants per group in a repeated-measures design would result in sufficient power (0.80) to detect significant differences (α-level = 0.05, *f* = 0.2). The study was carried out in accordance with the Helsinki Declaration of 1975 and its later revisions. Written informed consent was obtained from each athlete and there was no compensation for participation. The study was approved from the lead institution’s ethics board.

### Apparatus

**Figure 1:**
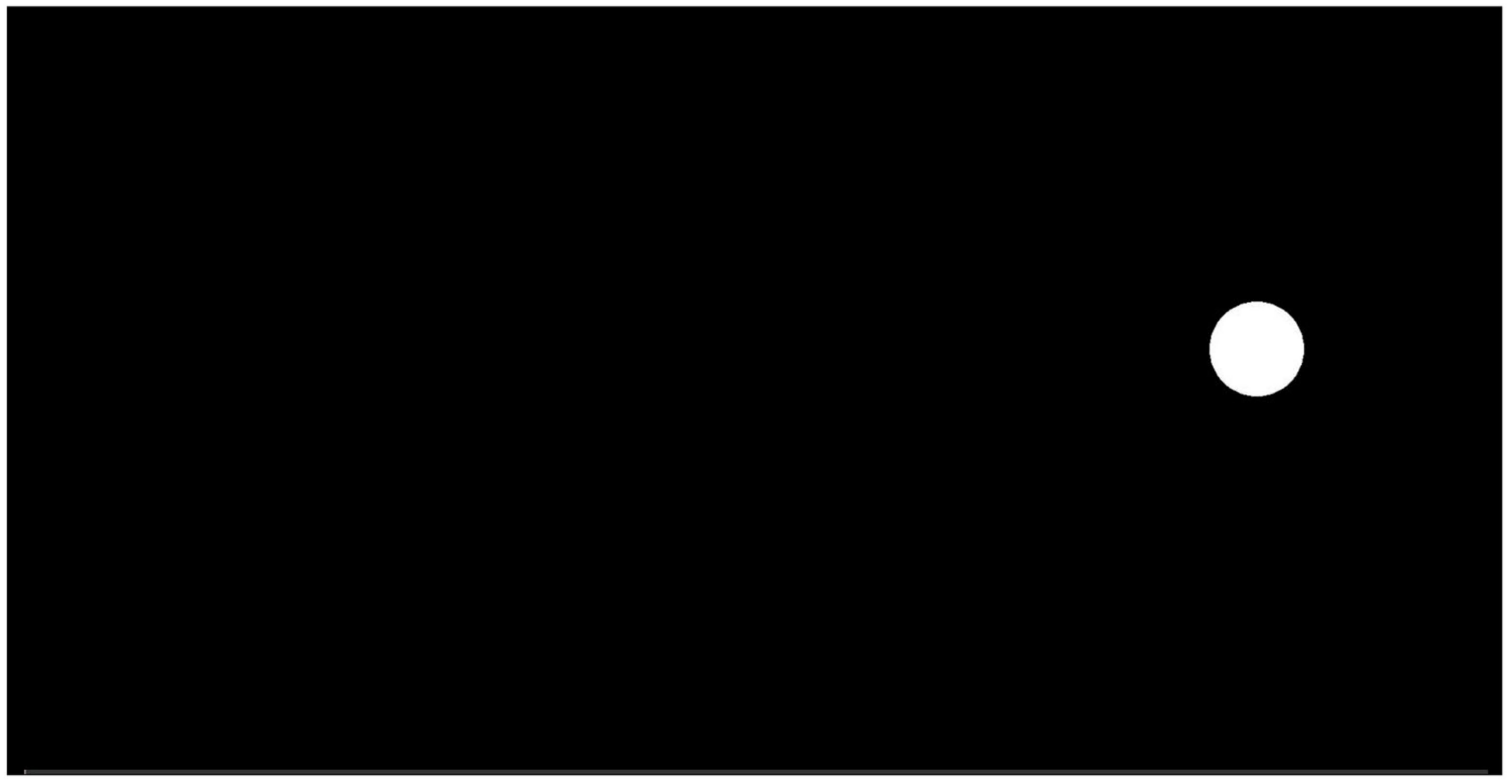
Picture of the object-tracking task. A white dot moved within the window for 60s and had to be visually tracked by the participants.

A visual object-tracking task was programmed in which a white ball moved randomly within a black window and bounced off the respective edges (Figure 1) for 1 min. At the beginning, the ball was positioned in the center of the screen before moving across the entire surface. The test was displayed on a 15″ screen. Furthermore, the participants also had to indicate on scales ranging from −10 to +10 how they felt with regard to an issue or problem that was troubling them in their career/life as a team sport athlete. This wellbeing-score before and after the intervention is part of the standard wingwave® approach. More important, it was also used in previous studies that focused on the use of the wingwave® method (e.g., Weiland et al., 2021). Using the same scale, therefore, allows to compare current and previous findings in a more straightforward way. Furthermore, the Pupil Lab’s® eye tracker Pupil Core® was used to detect gaze direction and gaze movements throughout the visual object-tracking task. The device worked with a 5 point calibration and a frequency of 200 Hz (192 × 192 pixels at 200 frames per second). It was linked to a laptop *via* USB and running with Pupil Capture® and Pupil Player® on the software side.

This eye tracking system has been applied in several previous studies including team sport athletes (e.g., Fasold et al., 2021; Klatt et al., 2021a, b). The reviews published by Kredel et al. (2017) and Hüttermann et al. (2018) provide a general overview of the use of eye tracking systems in sports.

### Coachings

Wingwave® coachings were performed by seven trained coaches of the wingwave® method who had at least 3 years of experience in coaching athletes. The decision about which coach would take over which coaching was based on the availability of the coaches at the respective test times, but each coach covered at least two coaching sessions. Coaches were advised to coach as they normally would but to first let participates introduce their problem. After that, they should manually test (see Rathschlag and Memmert, 2014) if the in-question problem is real and suitable as a starting point for a coaching session. In case a problem should not have been sufficient to serve as a starting point, a statement tree was prepared to give an overview of different types of possible problems. This statement tree would be used to clarify any issues/problems the participants had with other aspects of their team sports life. However, in all the cases, the participants’ self-reported problems were considered suitable as a starting point for wingwave® interventions so the statement tree was not used.

### Procedure

The subjects were first required to sign an informed consent form and then complete a questionnaire on demographic data (age, gender, team sports experience). They were then randomly assigned to either the wingwave® group or the control group, with 26 subjects (12 male, 14 female) in each of the groups. The allocation of the subjects was carried out by randomly assigning each subject to one of the groups. The first group participated in a coaching that lasted between 1 and 2 h. In the meantime, the other group watched a tennis match that was filmed from the side. This condition was chosen because it affords participants to move their eyes horizontally (as during a wingwave® session) but without a coach or other aspects of wingwave® coachings present. The length of this video was based on the length of the eye movement parts in the wingwave® coaching sessions. Before and after the coachings respectively, the control group treatment participants took part in the object-tracking task during which they had to think about their pre-existing problem and provided the severity of their team sport related problem on a scale ranging from −10 (as worse as possible) to +10 (no problem at all).

### Analysis

Individual ratings of the participants’ sport related issues were analyzed by a 2 × 2 mixed design ANOVA with group (wingwave®/control) as between-subject factor and time of testing (prior to/after coaching/control group treatment) as within-subject factor. To test for changes in the fluidity of gaze, videos of the eye tracking devices were analyzed for every participant and test session (prior to an after coaching/control group treatment). For this, similar to the procedure used by Kapoula et al. (2010), catch-up saccades were detected as measurement of fluidity of gaze (i.e., the less catch-up saccades made, the more fluid gaze tracking behavior). In our study, we defined a catch-up saccade as a deviation of a minimum of three degrees, followed by a rapid correction of gaze. The detection of the catch-up saccades was done by a manual frame-by-frame inspection of the recorded task. Afterwards, the number of catch-up saccades was analyzed by conducting another 2 × 2 mixed design ANOVA with group (wingwave®/control) as between-subject factor and time of testing (prior to/after coaching/control group treatment) as within-subject factor. In case of both ANOVAs, only/mainly the interaction effect was relevant for answering the research question, i.e., in how far both groups differently developed over time. Thus, interaction-effects were analyzed to evaluate whether the intervention affected participants’ individual ratings and gaze behavior differently than the control group treatment.

## Results

**Figure 2:**
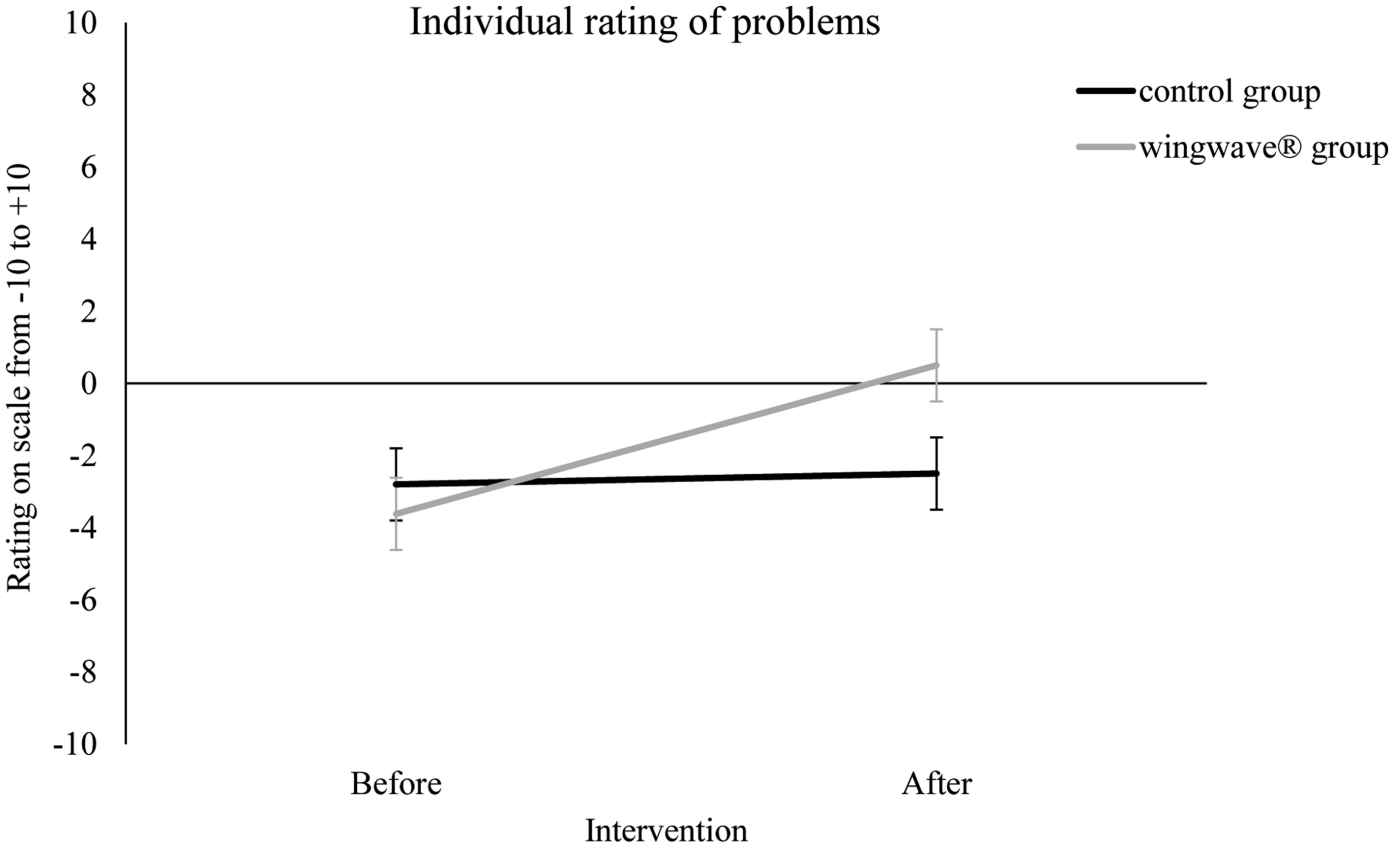
Individual ratings of team sport related problems as a function of treatment group (wingwave^®^, control) and moment of testing relative to intervention (before, after). Error bars indicate SEs.

Results of the ANOVA on individual ratings showed that the participants benefited more from a wingwave® coaching than from control group treatment, *F*(1, 50) = 22.428, *p* < 0.001, *eta^2^* = 0.310 (Figure 2). That is, whereas mean rating of the control group remained relatively constant (−2.81 prior to control group treatment, −2.5 afterwards) ratings of the wingwave® group improved from −3.62 to 0.5. Importantly, ratings between groups did not differ at the beginning of the study, *t*(50) = 0.891, *p* = 0.377.

**Figure 3:**
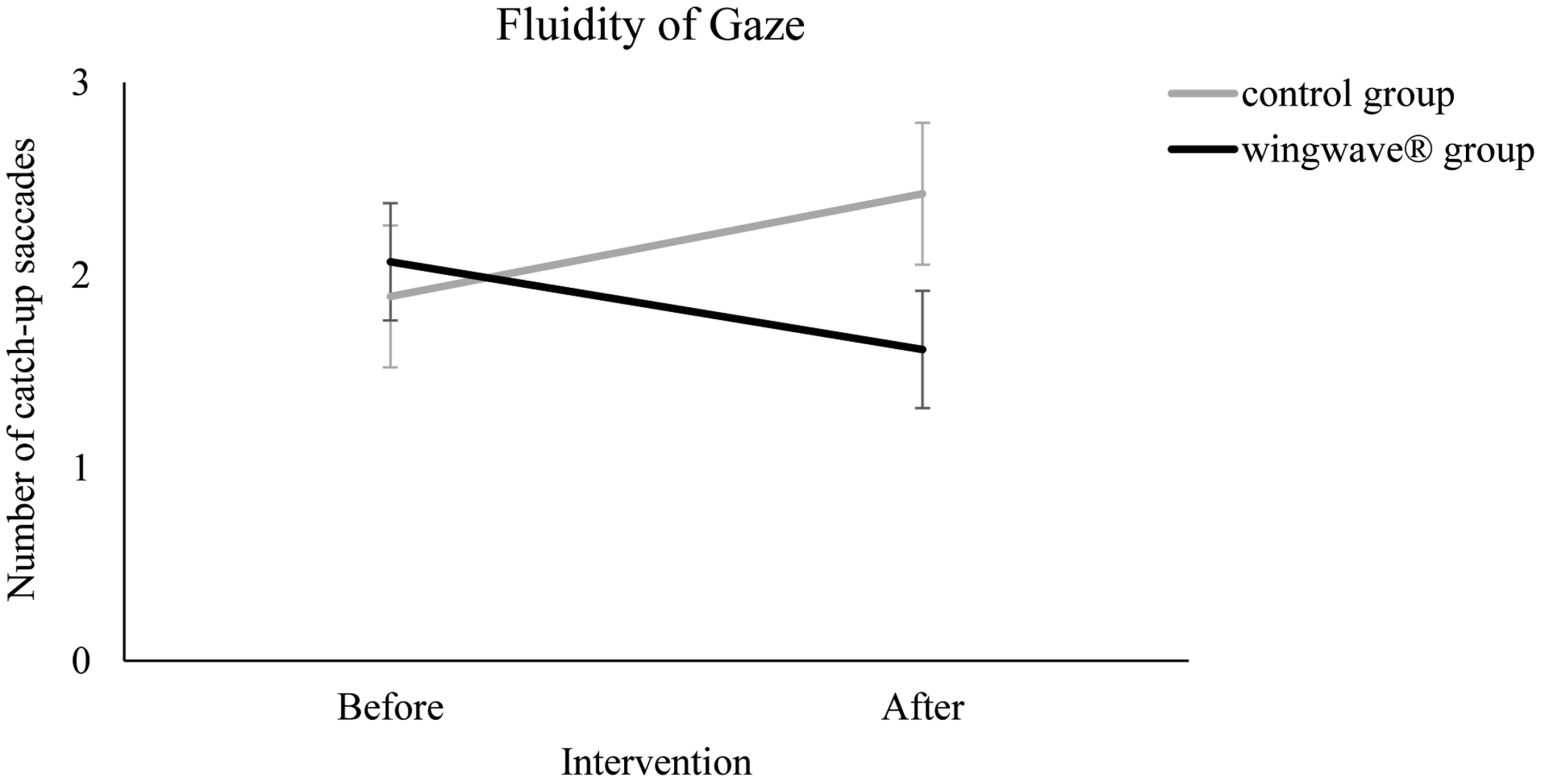
Fluidity of gaze measured as the number of catch-up saccades made during the object-tracking task as a function of treatment group (wingwave^®^, control) and moment of testing relative to intervention (before, after). Error bars indicate SEs.

Results of the second ANOVA showed that the fluidity of gaze as measured by the number of catch-up saccades increased in the wingwave® group (2.08 prior to control group treatment, 1.62 afterwards) and even got worse in the control group (1.88 vs. 2.42), *F*(1, 50) = 22.428, *p* < 0.001, *eta*^2^ = 0.310 (Figure 3). Again, ratings between groups did not differ at the beginning of the study, *t*(50) = −0.447, *p* = 0.657.

## Discussion

Given the high need of short-term interventions reducing anxiety in team sports athletes, we aimed to test whether wingwave® coachings efficiently reduce athletes’ ratings of sport related problems and whether such an improvement can be associated with changes in gaze (i.e., fluidity of gaze). Results showed that the individual ratings of pre-existing problems got smaller (also relative to a control group) after only one wingwave® coaching lasting about an hour. Furthermore, these improvements were accompanied by an increase in the fluidity of eye movements.

Thus, the results of the current study suggest that wingwave® interventions seem effective in reducing self-reported negative emotions respective problems of team sport athletes. Ratings of pre-existing problems even left the negative scale range after the interventions in some cases. Though this does not automatically mean that athletes will consequently perform better after wingwave® interventions, it shows at least that they subjectively feel better with regard to a pre-existing sport-related problem. However, an assessment of the athletes’ ability to cope with the challenge that they had previously faced and an assessment of the extent to which the wingwave® method impacted the change noticed, would have been desirable. As this was a limitation in this study, future studies could consider athletes with similar problems in different or same sports to further investigate generalizable results to the extent that meets the requirements of meaningful data. In the school context, Weiland et al. (2021) listed some typical cases: In the case of athletes in this study, there were mainly problems in the areas of (1) fear of injury, (2) fear of performing and/or competing, and (3) stressful memories of past competitions. Even though adding gender as a factor did not change the pattern of results in this study, it could nevertheless be of interest for future studies concerning the effect of a wingwave® coaching on the individual wellbeing.

There is evidence showing that negative emotions *per se* are not beneficial for performing well in competitive sports (e.g., Vast et al., 2010). It is, therefore, also an interesting topic for future studies to show if wingwave® interventions also affect behavior and performance in sport environments. It would then be especially easier to measure and for many athletes and coaches, could prove to be more decisive to focus on quantifiable performance in a sport before and after the wingwave® coaching including a control group. This could lead to a detection of noticeable changes in performance like Weiland et al. (2021) could show in the school context. Nevertheless, especially outside of professional sports, the wellbeing of athletes should be considered an end to itself. So, regardless the question if wingwave® coaching affects performance, it seems to offer a possibility to improve the personal wellbeing of athletes quickly or to reduce sports-related problems. Based on the recommendation Weiland et al. (2021) gave regarding the school-setting, wingwave® coachings brought to athletes from experts who work around individual athletes or teams could improve the performance environment and ease conflicts that arise throughout a season. This could be valuable given a rather low threshold (i.e., non-therapeutic) and fast approach (i.e., 1–2 h of coaching), based on the results of this study. Whether physicians, mental coaches, psychologists, or other professionals in this field apply the wingwave® method, is irrelevant compared to the recommended experience in dealing with it, its capabilities, and its limitations (e.g., it is a coaching method in a setting with mentally healthy clients and not a substitute for psychotherapy). Furthermore, the method is unfamiliar to most athletes and therefore, needs elucidation. Thus, it should only be applied by professionals, who feel confident in its application. Whether a coaching-process with the wingwave® method could be an alternative for team-building processes in general or an accurate choice for an intervention in a situation, in which a very specific issue is present, should also be subjected to further research. The use of fewer coaches in total and more athletes per coach could lead to variable results because in a sports team, there is commonly one coach for a heterogeneous team full of players with different needs and personalities. To what extent, despite the heterogeneity of the players, success in coaching can still be achieved by a single coach and whether the selection of several different coaches would be helpful in comparison to that, would be an interesting approach for a future study.

## Conclusion

Wingwave® interventions did not only affect subjective ratings of sport-related problems, but also led, as in previous studies on EMDR applications (Kapoula et al., 2010), to an increased fluidity of gaze in a smooth pursuit tracking task. That was also the case in comparison to a task in which participants moved their eyes in a comparable way as during wingwave® sessions. That is, the improvement in fluidity of gaze does not only seem to be a consequence of training horizontal eye movements during coaching sessions but should have its origin in other aspects of wingwave® interventions probably similar to effects of EMDR application on gaze behavior. However, how exactly wingwave® interventions increase fluidity of gaze also remains for future work though it seems likely that not being/being less concerned with a pre-existing problem during the tracking task (participants had to think about their problem while performing the tracking task) can at least explain parts of the improvement.

## Data availability statement

The original contributions presented in the study are included in the article/supplementary material, further inquiries can be directed to the corresponding author.

## Ethics statement

The studies involving human participants were reviewed and approved by ethics committee of the German Sport University Cologne. The patients/participants provided their written informed consent to participate in this study.

## Author contributions

BN and SK developed the study concept. BN collected the data and performed the statistical analysis. BN and FW wrote the first draft of the manuscript and share first authorship. All authors contributed to the article and approved the submitted version.

## Funding

This project was supported by a grant from the Gesellschaft für Neurolinguistisches Coaching e.V., Germany.

## Conflict of interest

The authors declare that the research was conducted in the absence of any commercial or financial relationships that could be construed as a potential conflict of interest.

## Publisher’s note

All claims expressed in this article are solely those of the authors and do not necessarily represent those of their affiliated organizations, or those of the publisher, the editors and the reviewers. Any product that may be evaluated in this article, or claim that may be made by its manufacturer, is not guaranteed or endorsed by the publisher.
